# Integration of Entrustable Professional Activities with the Milestones for Emergency Medicine Residents

**DOI:** 10.5811/westjem.2018.11.38912

**Published:** 2018-11-30

**Authors:** Danielle Hart, Douglas Franzen, Michael Beeson, Rahul Bhat, Miriam Kulkarni, Lorraine Thibodeau, Moshe Weizberg, Susan Promes

**Affiliations:** * Hennepin County Medical Center, Department of Emergency Medicine, Minneapolis, Minnesota; † University of Washington, Department of Emergency Medicine, Seattle, Washington; ‡ Akron General, Department of Emergency Medicine, Akron, Ohio; § MedStar Georgetown University Hospital, Washington Hospital Center, Department of Emergency Medicine, Washington, District of Columbia; ¶ St John’s Riverside Hospital, Department of Emergency Medicine, Yonkers, New York; || Albany Medical Center, Department of Emergency Medicine, Albany, New York; # Northwell Health, Department of Emergency Medicine, New York City, New York; ** Penn State Health, Department of Emergency Medicine, Hershey, Pennsylvania

## Abstract

**Introduction:**

Medical education is moving toward a competency-based framework with a focus on assessment using the Accreditation Council for Graduate Medical Education Milestones. Assessment of individual competencies through milestones can be challenging. While competencies describe characteristics of the person, the entrustable professional activities (EPAs) concept refers to work-related activities. EPAs would not replace the milestones but would be linked to them, integrating these frameworks. Many core specialties have already defined EPAs for resident trainees, but EPAs have not yet been created for emergency medicine (EM). This paper describes the development of milestone-linked EPAs for EM.

**Methods:**

Ten EM educators from across North America formed a consensus working group to draft EM EPAs, using a modified Glaser state-of-the-art approach. A reactor panel with EPA experts from the United States, Canada and the Netherlands was created, and an iterative process with multiple revisions was performed based on reactor panel input. Following this, the EPAs were sent to the Council of Residency Directors for EM (CORD-EM) listserv for additional feedback.

**Results:**

The product was 11 core EPAs that every trainee from every EM program should be able to perform independently by the time of graduation. Each EPA has associated knowledge, skills, attitudes and behaviors (KSAB), which are either milestones themselves or KSABs linked to individual milestones. We recognize that individual programs may have additional focus areas or work-based activities they want their trainees to achieve by graduation; therefore, programs are also encouraged to create additional program-specific EPAs.

**Conclusion:**

This set of 11 core, EM-resident EPAs can be used as an assessment tool by EM residency programs, allowing supervising physicians to document the multiple entrustment decisions they are already making during clinical shifts with trainees. The KSAB list within each EPA could assist supervisors in giving specific, actionable feedback to trainees and allow trainees to use this list as an assessment-for-learning tool. Linking each KSAB to individual EM milestones allows EPAs to directly inform milestone assessment for clinical competency committees. These EPAs serve as another option for workplace-based assessment, and are linked to the milestones to create an integrated framework.

## INTRODUCTION

Postgraduate medical education (GME) programs in the United States (U.S.) are moving toward a competency-based medical education (CBME) framework. In this system, GME programs will ensure that trainees demonstrate competence across the full spectrum of specialty-based work activities required to independently provide safe, quality patient care.

### Milestones, KSABs, Competencies and Competence

In 2012, the Accreditation Council for Graduate Medical Education (ACGME) in conjunction with the American Board of Emergency Medicine (ABEM) released the emergency medicine (EM) Milestones as a framework for training programs to guide the development of assessment of trainees’ progress towards competence in each domain.^1^ There are 23 domains, known as “sub-competencies” within the EM Milestones, each residing within one of the original six “core competencies” (medical knowledge [MK], patient care [PC], interpersonal and communication skills [ICS], professionalism [PROF], systems-based practice [SBP], and problem-based learning and improvement [PBLI]).^2^,^3^ Demonstrating “competence” in all of these milestone “sub-competencies” is required for graduation into unsupervised practice. Competence is defined by the Merriam-Webster dictionary as “the state of being competent,” and competent is defined as “having requisite or adequate abilities or qualities.” Each sub-competency is divided into five developmental levels (levels 1–5, also known as proficiency levels), containing descriptors of knowledge, skills, attitudes and behaviors (KSAB) appropriate for each level ranging from novice to expert provider. Each individual descriptor of the trainee, their KSABs, or their performance at a particular developmental level is known as an individual milestone (Figure). Individual milestones describe the KSABs required to progress from novice (level 1) to competent (level 4); they also detail a higher, aspirational level (level 5).^2^

### Entrustable Professional Activities and Observable Professional Activities

Entrustable professional activities (EPA) are observable, measurable, work-based activities. They have been defined as “units of professional practice that can be fully entrusted to a trainee, as soon as he or she has demonstrated the requisite competence to execute the activity unsupervised.”^4^ While competencies and milestones describe abilities or characteristics of the trainee (i.e., obtains an accurate and thorough history and exam, successfully performs intravenous line placement, communicates respectfully with patients), EPAs describe broader work-based activities (i.e., manages a critically ill patient).^4^–^7^ EPAs, when taken collectively, are “the essential professional activities that describe a specialty.”^5^

**Population Health Research Capsule**

What do we already know about this issue?

*Medical education is moving towards a competency based framework (CBME). Entrustable professional activities (EPAs) are one way to assess competence, and can be linked to the Milestones*.

What was the research question?

*To develop Milestone-linked EPAs for emergency medicine (EM) residents*.

What was the major finding of the study?

*Eleven core EPAs were developed for EM. Each EPAs has associated knowledge, skills, attitudes and behaviors (KSABs), which are linked to individual milestones or are milestones themselves*.

How does this improve population health?

*These EPAs give programs a method of workplace based assessment that may be more intuitive to use than milestones. Linking of KSABs to individual milestones allows for an integrated framework*.

The levels of EPA-related supervision are listed in Table 1.^4^–^6^,^8^ Since supervising physicians are already making decisions about how much supervision a particular trainee needs (in other words, how much they “trust” that trainee) multiple times per shift, EPAs may provide a more intuitive route to competency-based assessment.^4^,^9^ Since emergency departments (EDs) with trainees in the U.S. are staffed with attending physicians 100% of the time, making them generally “immediately available,” the Level 4 rating for EM trainees in the ED is more conceptual, with supervisors asking themselves, “Do I feel it would be appropriate for this trainee to perform this task if they were practicing independently, such as moonlighting at an external institution?” Level 5 may also not follow Level 4 sequentially in the ED, since senior residents supervising others are still supervised by an ED attending.

Observable practice activities (OPA) are defined by Warm et al. as “learning objectives/activities that must be observed in daily practice in order to form entrustment decisions.”^10^ OPAs are smaller units of directly observable practice than EPAs. Multiple OPAs are nested within each EPA, meaning that multiple OPAs would contribute to the entrustment decision for each larger EPA.

### Inter-relationship of the Competencies, Milestones, KSABs and EPAs

Most work-related activities require the integration of multiple competencies, sub-competencies and individual milestone items as well as some additional KSABs (Figure).^8^,^11^ For example, to decide that a trainee can manage a resuscitation with indirect supervision, the trainee must have previously demonstrated multiple KSABs described by the milestones within PC, SBP, ICS and PROF arenas. Thus, when assessing whether or not a trainee is capable of performing a work-based activity independently, the supervisor is indirectly deciding whether or not that trainee has attained those requisite milestones or competencies. EPAs and competencies are, therefore, inter-related. EPAs are not a replacement for the ACGME Milestones; rather they can be linked to individual milestones within their respective proficiency levels to create a unifying framework and provide more learner-centered information.^4^,^5^,^7^,^12^

### EPAs for Emergency Medicine

Many GME specialties are creating EPAs to augment their assessment landscape. Internal medicine (IM), family medicine (FM), psychiatry, radiology, anesthesia, pediatrics, and various fellowships have developed EPAs for their trainees.^11^,^13^–^24^ To our knowledge, EM EPAs have not yet been developed. We sought to develop EPAs for EM using a consensus process to encompass the full spectrum of work activities performed by emergency physicians in the cognitive and affective domains.^4^,^25^ We also aimed to link each EPA to the contributing, individual KSABs and milestone items, creating this unifying framework.^7^,^12^

## METHODS AND RESULTS

Glaser’s state-of-the-art approach to consensus has been recommended as an appropriate method for EPA development.^4^,^25^ We implemented Glaser’s approach to consensus in an iterative fashion with three modifications: 1) the group leader was a participant and a physician educator; 2) the consensus group members were not hand-picked by the group leader;^25^ and 3) not all members of this work group had experience with EM EPAs prior to this project. A group of 10 EM educators from across North America responded to a call for volunteers that was sent to the Council of Residency Directors for EM (CORD-EM) to serve on this work group to develop EPAs in EM. Using ten Cate’s recommendations, initial discussions centered around developing a guiding framework on which to structure the EPAs and to consider what work-based activities EM practitioners complete on a daily or weekly basis.^4^

To determine the content of the EPAs, the researchers drew from the Model of the Clinical Practice of Emergency Medicine and the EM Milestones.^26^,^27^ Given the broad scope of EM, a primary area of discussion was determining appropriate levels of focus and granularity. After the group initially considered writing EPAs for discrete patient complaints (similar to the approach taken by Shaugnessy et al. for FM), we realized this list would be too large and not sufficiently comprehensive. We also felt that narrowing the list of patient complaints through some form of nominal group technique would leave content gaps.^14^ This type of patient complaint-based assessment schema also seemed more consistent with OPAs, such as the 350 identified for IM.^10^ The granularity seen in sets of EPAs developed for less-advanced learners (i.e., medical or physician’s assistant students) also seemed inappropriate for resident trainees, because they would then not represent significant steps towards unsupervised practice, as recommended by ten Cate.^4^,^22^,^23^,^28^ Therefore, we made an a priori decision to broaden the scope of each EPA, with a goal of keeping the total number of OPAs to less than 30.^4^ We decided to develop examples of OPAs that would nest within each EPA, but not to develop a complete set of OPAs for this project.

We decided to exclude psychomotor procedural skills (including ultrasound) from our process, as the EM procedural milestones already can be used as a task-based assessment tool, and many other procedural assessment tools already exist. Further, entrustment decisions about individual procedures can be made independent of a trainee’s progress in other areas.

We also decided not to develop EPAs solely revolving around patient communication and professionalism. At the EM resident level of training, these do not represent an independent work-based activity separate from other aspects of a patient encounter. Communication and professionalism are intertwined into each patient encounter and are integral to many work-based activities, or EPAs. For example, a learner is not fully entrustable to care for a low-acuity, low-complexity “stable” patient unless they are able to communicate discharge instructions effectively to the patient. They are similarly not entrusted to manage a high-acuity, high-complexity patient unless they are able to effectively communicate with other healthcare team members, specifically nursing staff, ancillary staff, and consultants. Due to concerns that important professionalism and communication skills could get overlooked by assessors within these larger EPAs, we created a sub-section of EPA KSABs for ICS/PROF/SBP. We hope this will prompt assessors to recall the importance of accounting for these competencies in their overall EPA assessments.

Additionally, we decided not to create EPAs for performance improvement tasks such as creating one’s own performance improvement plan because, while extremely important, it would not make sense for a learner to only be allowed do this with close supervision until “entrustment,” precluding it from being a true EPA. Similarly, we did not create EPAs for wellness topics such as nutrition, exercise and psychological care because while these topics are important, they are not work-based activities nor must they be overseen until the trainee demonstrates competence; therefore, they should be assessed by different means. EPAs are not the mechanism to assess all personal aspects of being a good physician; they are solely intended to assess work-based activities.^4^

Over a period of approximately six months, using ten Cate’s recommended guidelines, we created a list of 29 EPAs.^5^ The list initially started with 19 EPAs, which was iteratively refined through multiple group meetings. Some EPAs were subdivided and additional new EPAs were suggested. We mapped the underlying KSABs to each EPA, and each KSAB was then mapped to the individual ACGME EM Milestone items. Level 5 milestones were generally excluded since these are not expected of trainees. We associated examples of OPAs, such as “manage acute coronary syndrome,” with each EPA to give the users a better understanding of what type of patient interactions or work-based activities would be included within each EPA.^10^

We formed a reactor panel of 15 individuals including EM program directors, thought leaders in EM education, and EPA experts from the U.S., Canada, and the Netherlands. All non-EM EPA experts (seven) had extensive experience with and previous publications on EPAs, and most EM experts (six) had extensive experience with and previous publications in medical education (reactor panel individuals are named in the “Acknowledgments” section). They suggested that several of the proposed EPAs be combined. The drafting panel revised the initial EPAs based on this expert feedback into a set of 11 EPAs. We returned the revised EPAs to the reactor panel for additional feedback and approval. We then sent this draft of 11 EPAs to the CORD-EM general membership listserv for additional comment and revision. Based on input from 61 respondents, subsequent minor revisions were made.

We feel that every trainee from every EM program should be able to perform these 11 core EM EPAs independently by the time they graduate to independent practice (Table 2, Appendix 1). Appendix 1 includes the details of the 11 core EPAs, including examples of patient presentations or situations (OPAs) that nest within each EPA, and the mapping of each EPA to the related milestones and KSABs. We have ensured that all milestone items within proficiency levels 1–4 have been mapped to KSABs within each EPA for all non-procedural patient care (PC1-8), interpersonal communication (ICS1-2), and systems-based practice (SBP1-3) sub-competencies. KSABs do not map to all level 1–4 milestones for MK, PBLI, and accountability (PROF2) as these milestones either primarily reflect qualities of the person or are not a work-based activity. This milestone in PROF2 “consistently recognizes limits of knowledge in common and frequent clinical situations and asks for assistance,” as well as a few others, are incorporated into our prerequisites for trust. Certain EPAs build on each other. For example, to achieve EPA #2 (managing a low-acuity, high-complexity “stable” patient), the learner must also have achieved the KSABs of EPA #1 (managing a low-acuity, low-complexity “stable” patient). These progressive EPAs are labeled as such in Appendix 1.

We also identified six baseline characteristics that are prerequisites to entrustment, meaning that a trainee would not be entrusted with any EPA until they have demonstrated these attitudes or behaviors (Appendix 2). As such, these are not included in the individual EPAs. These characteristics include three of ten Cate’s general conditions for trust: a) integrity, b) reliability, and c) humility, plus three additional factors: a) respectfulness, b) self-monitoring and resilience, and c) self-assessment and self-improvement. Ten Cate’s fourth condition for trust is ability, which is developed throughout residency and is addressed by our EPAs.^29^

For each EPA in Appendix 1, we provide five of the components of an EPA described by ten Cate: 1) title; 2) specifications and limitations; 3) relevant competency domains; 4) required KSAs; and 5) expected level of training for entrustment.^4^,^5^ Regarding “the expected level of training for entrustment,” our timeline is simply a suggestion, and different programs may adjust their own individual timelines to match their programmatic structure. We did not include ten Cate’s section of “expiration date.” This does not seem relevant to individuals still in residency programs, since EM trainees will continue practicing and demonstrating all of these skills for the entirety of their training.

We also did not include ten Cate’s “assessment information sources” section, because it should be left up to each program to determine how they can most feasibly and reliably assess each EPA. For all EPAs, when feasible, trainees should be observed in the clinical environment multiple times in varied contexts with a range of presenting patient complaints to ensure the trainee is able to reliably perform the EPA in differing circumstances. However, this is not always possible with less-common situations. Simulation and other sources such as standardized direct observation of training can also be used as contributing data sources.^30^ As with other competency decisions, no isolated assessment should result in a summative programmatic-level entrustment decision. This requires an integration of multiple data points or streams.^31^

We also recognize that individual residents may have specific areas of interest and individual programs may have specific areas of focus. Therefore, it would be appropriate for training programs to add program-specific or elective EPAs as appropriate for their specific setting, areas of focus or tracks, when available.^4^,^32^

## DISCUSSION

While milestones have moved us towards CBME in the U.S., the assessment of individual milestones has proven difficult, as evidenced by more programs than expected submitting straight-line scoring.^33^,^34^ This may be due to assessors having difficulty translating the level of trust they have for a trainee to perform a specific work-based activity into the multiple requisite competency domains. We hope that these EM EPAs may streamline this work-based assessment process.

EPAs could be more intuitive to assess than milestones because they capture assessment decisions that are already being made by supervising physicians dozens of times each shift.^9^ For example, with every patient, supervisors decide how much of the history and exam they need to confirm themselves, whether they need to double-check order entry or results, whether they need to be in the room for procedures or other patient-care related tasks, as well as other types of entrustment decisions. We are therefore not suggesting that EPAs replace milestones but rather should be used as a way of capturing assessment decisions in a format that is accessible to the learner, the supervisor and program leadership.

We also compiled baseline characteristics or competencies that are prerequisites to entrustment, instead of adding this list to each individual EPA. (Appendix 2).^29^,^35^ We feel these prerequisites are quite important, as without demonstrating these attitudes and behaviors the trainee should not be entrusted with any of the EPAs. For example, if a trainee lacks integrity and is not truthful or accountable for their actions and words, or lacks reliability in following through on tasks, the attending physician would not likely want to entrust them with any of the EPAs.

While part of the appeal of EPAs is their intuitive nature, we associated the requisite KSABs with each EPA for two reasons. First, if the supervisor is not comfortable with the learner performing the specific work activity (EPA) independently, the associated KSAB list can assist the supervisor in giving specific actionable feedback to trainees regarding what they need to work on in order to move towards the next level of entrustment. This allows the EPA to function as both an assessment of learning and an assessment-for-learning tool, allowing the program to gather data on which milestones are being met while assisting the learner in identifying areas that need further development.^36^ Second, in the U.S., we must report each trainee’s milestones to the ACGME bi-annually. Having each KSAB be an individual milestone, or be linked to an individual milestone, allows this assessment to directly translate into trainee progress in the milestones.

We recommend that, when possible, each EPA be assessed multiple times in various contexts with varying patient presentations and varied assessors. Our rationale for this is multifold. First, for example, regarding a low-acuity, low-complexity patient, one trainee may be entrusted to manage a patient with an earache but not a sore throat, or may be entrusted to manage a patient with lumbar but not thoracic back pain. Residents would need to be observed managing an array of low-acuity, low-complexity patients to ensure they should be entrusted to manage this type of patient independently or with distant supervision. This phenomenon may lead us to developing multiple OPAs for EM in the future, to nest within these overarching EPAs.^10^

Second, variables such as ED patient volume or internal or external stressors on the trainee may affect his or her ability to be entrusted with a certain task at various points in time. For example, a trainee may be able to manage a high-acuity, high-complexity patient in isolation, but when adding five other patients to care for concomitantly, the trainee may no longer be able to provide the level of care required to that high-acuity, high-complexity patient. Additionally, variables related to the supervisor may also impact the decision for entrustment in any one circumstance, such as internal or external stressors on the supervisor, the supervisor’s predilection for trust and risk tolerance, the relationship between the supervisor and the trainee, the amount of time the supervisor has spent observing the trainee previously, and the expertise the supervisor has in clinical and assessment arenas.^6^,^37^

Having some of the EPA KSABs describe performance expectations differently than the exact milestones allows these KSABs to serve as a complementary learning tool for trainees. EPA-labeled milestone assignments viewed by the clinical competency committees (CCC) may provide both CCCs and learners with more information, such as seeing that the learner is able to meet certain milestones for lower-acuity patients but not higher-acuity ones. This could allow improved coaching or goal generation for subsequent shifts.

Thus far, this group has developed and collected content validity evidence for this set of EM EPAs. Internal structure, response process, and relations to other variable validity evidence has not yet been evaluated. This requires further study. It is possible that subsequent validity testing could lead to future revision of these EPAs, addition of separate EPAs, or development of OPAs. While the breadth of each EPA may initially be concerning for a lack of specificity and utility, the specificity of the included KSAB/milestone lists within each EPA should make this useful to both the learners and the residency programs. Our group had significant debate about “lumping vs. splitting” and the level of granularity that should be encompassed by each EPA. In discussions with ten Cate and other EPA experts within our reactor panel, it was suggested that we opt for a lower level of granularity so that each EPA represents a significant “unit of EM practice” and a significant step toward increased entrustment for unsupervised practice.^4^

## CONCLUSION

This set of 11 core, EM resident EPAs can be used as an assessment tool by EM residency programs, allowing supervising physicians to document the multiple entrustment decisions they are already making during clinical shifts with trainees. The KSAB list within each EPA could assist supervisors in giving specific actionable feedback to trainees and allow trainees to use this list as an assessment-for-learning tool. Linking each KSAB to individual EM milestones allows EPAs to directly inform milestone assessment for CCCs. These EPAs serve as another option for programs to use for workplace-based assessment and are linked to the milestones to create an integrated framework.

## Supplementary Material





## Figures and Tables

**Figure:**
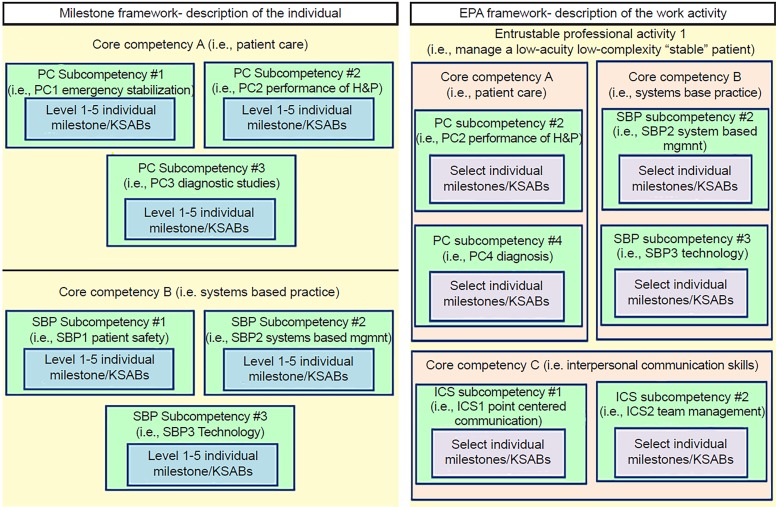
Milestone and EPA frameworks. *EPA*, entrustable professional activity; *KSAB*, knowledge, skills, attitudes, behaviors; *PC*, patient care; *H&P*, history and physical examination; *MK*, medical knowledge; *ICS*, interpersonal and communication skills; *PROF*, professionalism; *PBLI*, problem based learning and improvement; *SBP*, systems based practice; *mgmt*, management.

**Table 1:** Entrustable professional activity (EPA) levels.

EPA level	Description
Level 1	Trainee is not allowed to perform the activity at all.
Level 2	Trainee is allowed to perform the activity with direct supervision (supervisor present and proactive in the room).
Level 3	Trainee is allowed to perform the activity with indirect supervision (supervisor not present but is immediately available if needed).
Level 4	Trainee is allowed to perform the activity independently (with distant supervision not immediately available).
Level 5	Trainee is allowed to provide supervision to junior learners doing the activity.

**Table 2:** Core emergency medicine entrustable professional activities.

Manage a low-acuity, low-complexity “stable” patient. Manage a low-acuity, high-complexity “stable” patient. Manage a potentially high-acuity complaint in a “stable” patient. Manage a high-acuity patient with a well-defined presentation, illness, or injury. Manage a high-acuity, high-complexity patient (i.e., the undifferentiated unstable patient). Manage multiple patients in the emergency department (ED) concomitantly. Lead an ED team. Transition patient care to other healthcare providers. Manage interactions with consultants. Manage complex and difficult situations. Use recommended patient-safety and quality improvement processes.
