# Supplementary material for: Integration of Entrustable Professional Activities with the Milestones for Emergency Medicine Residents
Source: West J Emerg Med. 2018 Nov 30;20(1):35–42. doi: 10.5811/westjem.2018.11.38912 (PMC6324698; doi:10.5811/westjem.2018.11.38912)
Supplement: Supplementary file 1 [file wjem-20-35-s001.docx]

**Appendix 1.** Emergency Medicine EPAs.

1. **Title: Manage a low acuity, low complexity “stable” patient**.

Specifications and Limitations: This activity includes evaluating and managing patients who have common emergency department (ED) complaints often of low acuity, along with developing an appropriate disposition. This includes considering and inquiring about historical / exam factors that increase the likelihood of sinister pathology, recognizing when the patient does not fit into the usual algorithms, broadening or adjusting the workup and treatment plan when appropriate, and sufficiently documenting medical decision making (MDM). This also includes communicating with patients in a clear, effective, compassionate, and respectful manner.

Example observable practice activities (OPA’s) include evaluating and managing the following types of patients:

- Low back pain
- Ankle pain
- Sore throat
- Cough
- Rash

Most Relevant Domains of Competence, including Milestone Subcompentencies that map to this EPA:

- PC 2,3,4,5,6,7
- ICS 1,2
- PROF1,2
- SBP 2,3

| **Required KSA and behaviors. To fulfill this EPA, the Trainee must:** | **Milestone Mapping** |
| --- | --- |
| **Data gathering / Decision making** |  |
| Perform a reliable and thorough yet focused H&P, addressing chief complaint and urgent patient issues, including asking questions and performing exam elements to investigate the likelihood of potentially sinister pathologies | PC2.L1, PC2.L2, ICS1.L1b, 2a, PC4.L2a/b |
| Ascertain all relevant historical & exam details needed to develop an appropriate management plan, including information present in the EHR essential to patient care (i.e. vital signs, triage / RN notes, PMH, meds, allergies, prior clinic / ED visits) | PC2.L1,2,4, PC8.L1b,2b |
| Develop a broad differential diagnosis including potential serious etiologies and then narrow and rank appropriately based on information gathered | PC4.L1,2a/b,3a,4 |
| Recognize when a patient presentation does not fall into the usual algorithm and requires an alternate workup or treatment plan | PC4.L5 |
| Develop, present and commit to an initial diagnostic / management plan, including recalling and/or accessing medical information, including clinical decision rules and other EBM strategies to determine need for testing (i.e. Ottawa ankle rule) | PC3.L1, 2a/b, 3a, 4a, PBLI.L3c |
| Develop, present and commit to an entire longitudinal ED plan (including IF-THEN statements), including anticipating and preparing for disposition | N/A, PC7.L3c |
| Demonstrate accurate and timely order entry | PC8.L1, SBP3.L1a |
| Reassess patient at appropriate intervals including response to treatment, allowing this to guide further ED management, including broadening or modifying differential diagnosis when new information arises or data arises that doesn’t support the initial working diagnosis | PC4.L3, PC6.L2, 3b/c, 4 |
| Interpret results of tests correctly | PC3.L3b |
| Select appropriate medications to treat patient's condition, incorporating items such as patient allergies, current medications and local antibiotic resistance into decision, and incorporating EBM | PC5.L1a/b, 2a, 3a, PBLI.L3c, SBP3.L1b |
| Select appropriate non-pharmacological treatments and decide on appropriate management and follow up plan incorporating EBM | PC4.L4, PC7.L2, PBLI.L3c |
| Demonstrates reasonably cost effective utilization of resources including diagnostics and therapeutics, considers pre-test probability and the likelihood of test results altering management | PC3.L4a/b, SBP2.L3a |
| Develop specific discharge plans with appropriate outpatient follow up and return to ED precautions to optimize the patient’s overall healthcare, and implement these plans in a timely fashion | PC7.L1/2, PC8.L1 |
| Ensure all test results and vital signs are reviewed prior to discharge and recognize abnormal values & results | PC1.L1, PC3.L3b, PC6.L1, |
| **Communication / Professionalism / SBP** |  |
| Convey a genuine interest in the patient through use of verbal and non-verbal communication strategies and active listening skills, ascertains the true primary reason for the patient’s ED visit, and addresses patient and family concerns | PROF1.L1,2, ICS1.L1a,1b,2a |
| Recognize the importance of and addresses patient opinions, feelings and beliefs | PROF1.L1a/b |
| Give patient honest answers to questions, and is able to say “I don’t know” when appropriate | ICS1.L3a, PROF1.L1 |
| Demonstrate clear, effective, compassionate, and respectful communication with the patient using non-medical jargon. This also includes providing patient with realistic expectations about anticipated plan of care, keeping patient updated on progress / delays during ED stay, and providing clear written and verbal discharge instructions | ICS1.L1, 3a, SBP2.L2b, SBP3.L2a/b, PROF1.L1,2 |
| Ensure patient understands communication in ED, especially discharge instructions by using talkback or other accepted techniques | PC7.L4b |
| Communicate thoughts and plans concisely and accurately to the supervising physician | ICS2.L1,2 |
| Communicate with nursing and ancillary personnel including social work and financial aid as needed to optimize patient care | ICS2.L1,2,3a/c, SBP2.L1,2a, PC7.L3b |
| Complete documentation in order to 1) communicate thought processes and medical decision making to future healthcare providers in an accurate and complete yet concise manner, 2) describe what serious diagnoses where considered but not investigated for medicolegal purposes and 3) enable the chart to be billed at the correct level of service. | SBP3.L1a,2a |
| Ensure own documentation is accurate and free from errors | SBP3.L3 |
| *Apply service excellence models to enhance patient satisfaction where applicable* | *SBP2.L2b* |

At which stage of training are trainees expected to achieve Level 4 entrustment: early in PGY-2 year.

1. **Title:** **Manage a low acuity, high complexity “stable” patient**.

Specifications and Limitations: This activity includes evaluating and managing low acuity yet highly complex patients, including those with multiple or vague complaints, those whose medical complaints could be confounded by psychiatric issues, social issues or other motivators, those where historical information is limited, or those with a complicated PMH, and identifying those with the potential to have more serious pathology or do not fit into the usual algorithms. This also includes communicating in a way these patients or their caregivers can understand and to optimize their care during and after the ED encounter especially when uncertain of the diagnosis, ensuring patient safety, and sufficiently documenting the MDM.

Example observable practice activities (OPA’s) include evaluating and managing the following types of patients:

- A patient with multiple complaints, having the ability to ascertain and focus the workup around the most concerning complaint even if that was not the patient’s primary complaint, and not fully evaluating the entire multitude of complaints,
- A patient with vague complaints such as “feeling unwell” which varies based on patient age and comorbidities
- A patient with unusual or uncommon complaints such as intermittent episodes of flushing
- A patient who is unable to provide much history, such as those with prior aphasia or abnormal baseline mental status,
- A patient who has limited resources and health literacy and has difficulty with outpatient follow up,
- A patient who has a seemingly simple complaint but also has significant confounding variables such as a very complicated past medical history (PMH) & medication list or who is at extremes of age
- A patient who has a new serious but subacute diagnosis, such as cancer

Most Relevant Domains of Competence, including Milestone Subcompentencies that map to this EPA:

- PC 2,3,4,5,6,7
- ICS 1,2
- PBLI
- PROF1,2
- SBP 1,2,3
- MK (in general, not mapped to milestone levels)

| **Required KSA & behaviors. To fulfill this EPA, the Trainee must:** | **Milestone Mapping** |
| --- | --- |
| Perform all of the KSAs from EPA #1 in this context and do the following: | |
| **Data gathering / Decision making** |  |
| Ascertain data from other sources aside from the patient when needed and determine the accuracy of the data gathered from the patient versus those various sources (i.e. family members, bystanders, EMS crew, nursing home, state/regional prescription monitoring program, prior clinic / ED visits, etc.) | PC2.L4, PC4.L4, ICS1.L1b |
| Prioritize multiple complaints and recognize what needs workup in the ED versus what can be worked up as an outpatient in the clinic | PC2.L2,3a,3b, PC3.L4b, SBP2.L3a |
| Recognize limits of own knowledge or abilities and involve consultants when needed | SBP2.L2a,3b |
| “Recognize limits of knowledge in uncommon and complicated clinical situations; develops and implements plans for the best possible patient care when such uncertainty exists” | PROF2.L3a |
| Uses just-in-time resources / references when needed to guide patient care plans | SBP1.L3c |
| Consider potential side effects or drug interactions in medication selection | PC5.L2b,3b |
| Consider financial considerations, local resistance patters, patient age, weight, and other modifying factors into medication selection | PC5.L4 |
| Identify, navigate and communicate with the patient about social, psychiatric and financial confounders, involving social work, care coordinators, financial aid, etc. when appropriate | PC7.L3b, SBP2.L2a,3b, ICS1.L3b |
| “Makes correct decisions regarding admit versus discharge” | PC7.L3c |
| “Develop alternate treatment plans when patients have beliefs that preclude commonly accepted care” | PROF1.L3b |
| “Creates discharge plans to include future diagnostic and therapeutic interventions” | PC7.L4a |
| **Communication / Professionalism / SBP** |  |
| Reviews and discusses the plan of care with nursing staff when it deviates from usual practice or is not communicated well through orders alone | ICS2.L3a/c |
| Ensure safety of all patients by following patient safety recommendations (i.e. putting bedrails up when leaving room, check 2 for safety, etc.) | SBP1.L1a,2,3a/b |
| Ensure safety of high risk patients by following institutional protocols (i.e. getting a 1:1 for suicidal patients, etc.) | SBP1.L1,2,3a |
| Modify plans based on intrinsic patient factors when appropriate (i.e. ability to follow up, patient reliability, etc.) | PC7.L3a, ICS1.L3a/b |
| Recommend strategies in which patients’ access to care can be improved (i.e. realizing they don’t follow up b/c they don’t have transportation, helping them get clinic appointments in a more timely manner, etc.) | SBP2.L4b |
| Recognize when a patient or family is vulnerable (i.e. limited resources, limited health literacy, special needs), anticipates challenges, and develops plans for these patients in a manner that addresses their individual needs | PC7.L3a/b, SBP2.L2a,3b, |
| Communicate plans, including discharge plans, to the vulnerable patient to optimize understanding and follow through | ICS1.L3a/b, PC7.L3a,4b |
| Consistently prioritize the patient's best interests in all relationships and situations even when faced with barriers | PROF1.L4a |
| Utilize shared decision making when appropriate | NA |
| Deliver bad news to patients and families in an empathetic fashion, using generally accepted language and techniques | ICS1.L1a,3a,4a |

At which stage of training are trainees expected to achieve Level 4 entrustment: middle of PGY-2 year.

1. **Title: Manage a potentially high acuity complaint in a “stable” patient**

Specifications and Limitations: This activity includes evaluating and managing patients who have common ED complaints that could be indicative of higher acuity conditions, along with developing an appropriate disposition. This includes considering and inquiring about historical / exam factors that increase the likelihood of sinister pathology, recognizing when the patient does not fit into the usual algorithms, broadening or adjusting the workup and treatment plan when appropriate, and sufficiently documenting medical decision making (MDM). This also includes communicating with patients in a clear, effective, compassionate, and respectful manner.

Example observable practice activities (OPA’s) include evaluating and managing the following types of patients:

- Chest pain
- Shortness of breath
- Abdominal pain
- Headache

Most Relevant Domains of Competence, including Milestone Subcompentencies that map to this EPA:

- PC 1,2,3,4,5,6,7
- ICS 1,2
- PROF1,2
- SBP 2,3

| **Required KSA & behaviors. To fulfill this EPA, the Trainee must:** | **Milestone Mapping** |
| --- | --- |
| Perform all of the KSAs from EPA #1 in this context and do the following: | |
| **Data gathering / Decision making** |  |
| Prioritizes urgent diagnostics on these patients with potentially concerning etiologies (i.e. EKG for CP, CXR or US for suspected PTX, etc.) | PC3.L2a,3a |
| Prioritizes urgent therapeutics on these patients (i.e. nebs for asthma with moderate respiratory distress) | PC1.L2a,3a |
| **Communication / Professionalism / SBP** |  |
| Communicates pertinent information to the patient's nurse and other relevant team members, such as when certain diagnostics / therapeutics need to be to prioritized | ICS2.L1,2,3a,3c, SBP2.L1,2a |
| Demonstrates the ability to use resources in the system effectively to provide optimal and expedient health care (i.e. calls for a portable CXR if needed instead of sending pt. to radiology, etc.) | SBP2.L2a,3b |

At which stage of training are trainees expected to achieve Level 4 entrustment: middle of PGY-3 year

1. **Title: Manage a high acuity patient with a well-defined presentation, illness, or injury**

Specifications and Limitations:

This activity includes evaluating and managing patients who are known to be high acuity prior to or on arrival to the Emergency Department with a well-defined medical, traumatic, or psychiatric presentation (i.e. cardiac arrest, trauma, STEMI, stroke, acute psychosis). Although these patients can be very complex, initial management is often protocol driven (ACLS, ATLS, hospital STEMI or stroke pathway, etc.). Initial steps in patient care must be initiated expeditiously in parallel with prioritizing essential diagnostics and therapeutics, rapidly interpreting and incorporating results, and reassessing the patient in order to broaden or modify the differential diagnosis or plan of care as the clinical situation evolves, and involve appropriate consultants and hospital resources. The resident must maintain leadership of the resuscitation team, communicating with and directing team members (including consultants) effectively and delegating responsibility appropriately. They must display situation monitoring / situational awareness and address changes in team function or patient status expeditiously, providing redirection when needed. The resident must also demonstrate effective, compassionate, timely and respectful communication with the patient/family.

*Although performing individual procedures is not formally a part of this EPA, as team leader, the resident must be able to perform all key procedures competently themselves if necessary.

Example observable practice activities (OPA’s) include evaluating and managing the following types of patients:

- A patient who meets system trauma criteria
- A medical resuscitation of known etiology (e.g. v-fib arrest)
- An acute behavioral emergency / combative patient
- A “code stroke” patient
- A STEMI patient
- A patient in active labor / with precipitous delivery

Most Relevant Domains of Competence, including Milestone Subcompentencies that map to this EPA:

- PC1, 2, 3, 4, 5, 6, 7
- ICS 1,2
- SBP 1,2

| **Required KSA & behaviors. To fulfill this EPA, the Trainee must:** | **Milestone Mapping** |
| --- | --- |
| **Data gathering / Decision making / Patient Management** | |
| Recognize the “sick” or unstable patient | PC1.L2a, PC4.L3b |
| Performs a primary assessment (i.e. ABCs) | PC1.L2b |
| “Prioritize initial stabilization and management actions in the resuscitation of a critically ill or injured patient” | PC1.3a/b |
| Know and utilize resuscitation algorithms (ACLS, ATLS, etc.), institution-specific protocols (STEMI pathway), and other medical information to inform workup & management plan | PBLI.L3c |
| “Reassess after implementing a stabilizing intervention” | PC1.L3c |
| Prioritize essential components of the history & physical exam, while appropriately prioritizing interventions which may need to supersede history and/or exam | PC1.L2c,3b, PC2.L2,3a/b,4 |
| Appropriately utilize additional sources to gather essential information (e.g. EMS report, EHR review, family, etc.) | PC2.L4 |
| “Prioritize essential testing” | PC3.L1,3a |
| Prioritizes diagnostics and therapeutics appropriately throughout the case (i.e. does not go to head CT prior to getting ABCs addressed) | PC3.L3a |
| Order correct medications and doses for common emergent situations | PC5.L2a |
| Interpret results of a diagnostic study, such as ECG’s, US, labs and radiographic imaging. | PC3.L3b |
| “Develop a list of ranked differential diagnoses including those with the greatest potential for morbidity or mortality” | PC4.3a |
| Modify/revise the differential diagnosis and treatment plan as new information becomes available or in response to changes in a patient’s course over time | PC4.3c  PC6.3b/4a |
| Uses just-in-time resources / references when needed to guide patient care plans | SBP1.L3c |
| Requests the most appropriate admitting team and correct level of inpatient care | PC7.L3c/d |
| “Know indications, contraindications, anatomic landmarks, equipment, anesthetic and procedural technique, and potential complications for common ED procedures” | PC9.2a/b/c |
| “Perform post-procedural assessment and identifies any potential complications” | PC9.L2b |
| “Determine a backup strategy if initial attempts to perform a procedure are unsuccessful” | PC9.L3a |
| **Communication / Professionalism / SBP** | |
| “Effectively communicate with the patient or family, using communication methods that minimize the potential for stress, conflict, and misunderstanding” | ICS1.L3a |
| Manage the expectations of the patient/family, including providing updates, likely next steps and potential prognosis | ICS1.L2b,3a |
| Use flexible communication strategies to deliver updates and lead difficult conversations, including delivering bad news, death notification, unexpected outcomes, end of life care, and code status discussions | ICS1.L4 |
| “Interpret advanced directives and DNR forms” | PC1.L3d |
| Communicate pertinent information, including condition updates and test results, to (supervising) emergency physicians and other healthcare colleagues | ICS2.L2 |
| Create a “shared mental model” among team members during the resuscitation | ICS2.L3a/c |
| Ensures clear communication (such as closed loop communication) between team members during resuscitation | ICS2.L3a/c, SBP1.L4c |
| Ensures respect among team members during resuscitation including receiving input / suggestions / questions from other team members in a collaborative fashion | ICS2.L3a/c |
| Display situation monitoring / situational awareness and address changes in team function or patient status expeditiously, providing redirection when needed | SBP1.L4c |
| Ensures communication is clear between teams (i.e. emphasizes most relevant aspects early in discussion (i.e. reason for admission / consultation) during handoff / transition of care or during consultation, yet provides all important details) in an efficient manner | ICS2.L3b |
| “Call effectively on and coordinate additional resources (such as consultants, chaplains, etc.) in the system to optimize the patient’s care” | PC1.L4b, SBP1.L3c, SBP2.L4c |
| Appropriately use system resources to improve patient care, including consulting teams at appropriate times with appropriate levels of urgency (i.e. trauma *1 vs *2) | PC1.L4b, SBP1.L3c, SBP2.L4c |
| If discrepancy between consulting team and resident’s plan, the resident discusses and discovers the reason for the discrepancy | ICS2.L4b |
| “Identify and correct situations when the breakdown in teamwork or communication may contribute to medical error or diminished patient care quality” | SBP1.L4c |

At which stage of training are trainees expected to achieve Level 4 entrustment: late PGY2/early PGY3 year

1. **Title: Manage a high acuity, high complexity patient (i.e. undifferentiated unstable patient).**

Specifications and Limitations: This activity includes instituting initial resuscitation steps, data gathering, and prioritizing initial diagnostics for a patient with pathology that is initially unknown, unclear or confusing. Formulating a broad initial differential diagnosis to guide the overall patient care plan even though uncertainty remains is essential, followed by modifying this differential and treatment plan based on additional information gathered, results of diagnostics, and patient response to treatment. Overall patient / family and team communication, team leadership and situational awareness is essential (as above); prioritizing desired actions for nursing and consultants and maintaining control in complicated resuscitations is also imperative. Developing alternate treatment plans when patients have beliefs that preclude commonly accepted care is also required, as is involving appropriate consultants and hospital resources.

* Although performing individual procedures is not formally a part of this EPA, as team leader, the resident must be able to perform all key procedures competently themselves if necessary.

Example Observable Practice Activities (OPAs) include evaluating and managing the following types of patients:

- An unstable trauma patient with multiple medical comorbidities
- An unstable trauma patient with multiple injuries leading to competing priorities
- An unstable patient with altered mental status (e.g. “found down”)
- An unstable patient with an unknown etiology (e.g. CC = “dizzy” and patient is hypotensive & bradycardic)
- An unstable patient with an unclear code status

Most Relevant Domains of Competence, including Milestone Subcompentencies that map to this EPA:

- PC 1,2,3,4,5,6,7
- ICS 1,2
- SBP 1,2

| **Required KSA & behaviors. To fulfill this EPA, the Trainee must:** | **Milestone Mapping** |
| --- | --- |
| Perform all of the KSAs from EPA #3 in this context and do the following: | |
| **Data gathering / Decision making** | |
| *“Recognizes in a timely fashion when further clinical intervention is futile”* | PC1.L4a |
| *“Interprets validity of DNR” or other healthcare directive forms* | PC1.L3d |
| *“Effectively analyzes and manages ethical issues in complicated and challenging clinical situations”* | PROF1.L4 |
| Utilizes additional sources to gather essential information (e.g. calls care facility, calls patient’s medical decision maker, sends police to patient’s house to gather essential information) | PC2.L3,4 |
| “Prioritize critical initial resuscitation / stabilization actions,” even in the face of uncertainty | PC1.L3b, PC3.L3a;  PROF2.L3a |
| Order correct medications and doses for less common emergent situations, or when modifying factors exist such as patient weight, age, etc., and considers possible or anticipated adverse side effects | PC5.L4 |
| Develop an appropriately broad differential diagnosis including those with potential serious etiologies | PC4.L3a |
| “Synthesize all of the available data and narrow/prioritize the list of weighted differential diagnoses to determine appropriate management” | PC3.L3b,4 |
| “Reviews risks, benefits, contraindications and alternatives to a diagnostic study or procedure” | PC3.L3c |
| “Recognize limits of knowledge in uncommon and complicated clinical situations; develops and implements plans for the best possible patient care” | PROF2.L3a |
| Anticipates evolution of patient condition and plans 2-3 steps ahead, including having back-up plans if initial intervention not successful (i.e. if NRB does not help hypoxia / increased WOB, plans to do bi-pap next; calls for PRBCs prior to patient arrival for GSW chest) | NA, PC9.L3a |
| Is able to think outside the box when the usual treatment strategies are not available or under extenuating circumstances (i.e. cath lab not available so give tPA & transfer) | PROF2.L3a |
| Understands limitations of testing, including the implications of false positives and false negatives in post-test probability | PC3.L4a/c |
| “Formulate a sufficient admission plan (or discharge instructions) including future diagnostic/therapeutic interventions for ED patients” | PC7.L4 |
| “Performs indicated procedures on any patient, including those with challenging features (e.g., poorly identifiable landmarks, at extremes of age or with co-morbid conditions)” | PC9.L1a/b,2a/b/c,4a |
| Performs indicated procedures, takes steps to avoid potential complications, has a back-up plan, and recognizes the outcome and/or complications resulting from the procedure | PC9.L3a,4b |
| **Communication / Professionalism / SBP** | |
| Communicate with nursing about the planned next few steps and priorities of multiple actions | ICS2.L2,3c |
| Coordinate the input of multiple consulting teams & activities when patient management requires a multidisciplinary approach, ensuring maintaining overall resuscitation priorities (i.e. ABCS prior to isolated orthopedic procedures) | PC1.L4b,5a |
| “Uses flexible communication strategies to resolve difficulties with consultants, while prioritizing the patient's best interests” | ICS2.L4b |
| The resident must recognize when additional resources are needed and utilize them appropriately, but not over utilize resources and consultants prior to identifying a true need. | PC1.L4b, SBP2.L2a,3b,4c |
| Owns the resuscitation room with multiple consultants and in chaotic situations | SPB1.L4, SBP.L4c, ICS2.L4a/b |
| Leads team debriefing following challenging cases in order to improve future ED performance | SBP1.L4b |

At which stage of training are trainees expected to achieve Level 4 entrustment: end of PGY3 year

1. **Title: Manage multiple patients in the ED concomitantly**

Specifications & Limitations: The resident is able to care for multiple patients (i.e. 4-5) simultaneously, demonstrating an appropriate balance of thoroughness and efficiency for individual patients, efficient task switching, timely and accurate order entry, timely patient reassessments and subsequent decision making, and timely disposition planning and dispositions. Is able to prioritize tasks appropriately for optimal patient flow as well as identify “sick” patients and initiate care for them expediently. This EPA revolves around flow and prioritization and does not encompass the decision making or management with respect to each individual patient.

Example Observable Practice Activities (OPAs): NA

Most Relevant Domains of Competence, including Milestone Subcompentencies that map to this EPA:

- PC 1,2,6,8
- SBP 2
- ICS 2

| **Required KSA & behaviors. To fulfill this EPA, the Trainee must:** | **Milestone Mapping** |
| --- | --- |
| **Individual patient flow** |  |
| Evaluates patients in a timely manner after being roomed | PC8.L1 |
| Places initial orders in a timely fashion after seeing the patient | PC8.L1, SBP3.L1a |
| “Ensures that necessary diagnostic and therapeutic interventions are performed during a patient’s ED stay” in a timely fashion | PC6.L2,3c |
| Notices when delays in patient care are occurring, investigates the problem, and works on solutions (i.e. notices the patient hasn't had his XR for an hour, and talks with patient's nurse about it) | PC8.L3, SBP.L2a,3b |
| Communicates with nurses and ancillary staff in a concise and accurate fashion at appropriate intervals throughout the patients ED course (i.e. early when additional services such as social work are needed, when delays in care are encountered, when patient status changes) | ICS2.L1,2,3a,3c, PC7.L3b & SBP2.L1,3b |
| “Monitors a patient's clinical status at timely intervals during a patient's ED stay” and evaluates effectiveness of therapies and treatments provided | PC6.L3b/ c |
| Is able to anticipate disposition early in most patient's courses and is able to start working towards that early in patient's ED course | PC7.L3c, PC8.L3 |
| Recognizes when patients will need re-evaluation including over longer intervals such as observation in the ED | PC6.L1,3a |
| Discharges patient in a timely fashion when discharge is appropriate & workup is complete | PC8.L2,3 |
| Enters admission order in a timely fashion when admission is appropriate and level of care is known | PC8.L2,3 |
| Manages and prioritizes (or delays) interruptions in a professional manner and is able to task switch when needed | ICS2.L3a, PC8.L2,3 |
| **Recognizing and prioritizing the “sick” patient** |  |
| Recognizes the “sick” patient who requires timely or urgent intervention including abnormal vital signs, and does not let triage cuing or diagnostic momentum impair their ability to identify these patients as "sick" | PC1.L1, 2a, PC4.L3b |
| Prioritizes caring for the "sick patient" and higher acuity patients over other patients / tasks | PC1.L3a, PC8.L1,2 |
| Communicates pertinent information about the severity / acuity of the “sick” patient to the patient's nurse and other relevant members of the healthcare team, and clearly communicates what diagnostics / therapeutics to prioritize | PC2.L3b, PC3.L3a, ICS2.L1,2,3a,3c, SBP2.L1,2a |
| Demonstrates the ability to use resources in the system effectively to provide optimal and expedient health care (i.e. calls for a portable CXR instead of sending pt. to radiology, etc.) | SBP2.L2a,3b, PC3.L2 |
| Informs patient and family of the urgency of the situation in a compassionate way | ICS1.L3a |
| Responds to urgent RN concerns in a timely fashion | ICS2.L3a |
| **Departmental / systems management** |  |
| Is able to task switch between "sick" patients and lower acuity patients (i.e. does not "camp out" in a critically ill patient's room) | PC8.L1,2,3 |
| Appropriately utilizes ancillary services to assist in patient care | SBP2.L2a |
| “Involves appropriate resources (e.g. PCP, consultants, social work, PT/OT, financial aid, care coordinators) in a timely manner” when required | PC7.L3b |
| Troubleshoots and develops workarounds for unanticipated difficulties in implementing plans (i.e. lab delays, getting equipment / supplies sent from other hospital locations, etc.) | SBP2.L3b, SBP2.L4a |
| Requests appropriate level of care for admitted patients | PC7.L3d |

At which stage of training are trainees expected to achieve Level 4 entrustment: middle of PGY2 year

1. **Lead an ED team**

Specifications & Limitations: This activity includes managing an entire ED team (can be an entire physical ED or a significant zone / pod / portion of the ED), managing flow and operations, including in surge situations. Demonstrating situational awareness and investigating changes in their environment, modifying or choosing particular patient care plans based on overall ED volume and flow when appropriate, tailoring one's personal workflow to needs of the overall ED and accounting for the needs of the team in decision making, providing pre-hospital medical direction, and potentially implementing solutions to bottlenecks or high volume situations is required. Maintaining an appropriate balance of thoroughness and efficiency during high volume situations is also imperative. In addition to the skill of leading tasks or work, the leader’s communication style should be respectful and professional, they should be a positive role-model, create an open and engaging work environment, and represent departmental goals and values.

This EPA does not include coordinating and running a large-scale Mass Casualty Incident.

Example Observable Practice Activities (OPAs): NA

Most Relevant Domains of Competence, including Milestone Subcompentencies that map to this EPA:

- PC8
- ICS2
- SBP2

| **Required KSA & behaviors. To fulfill this EPA, the Trainee must:** | **Milestone Mapping** |
| --- | --- |
| Perform all of the KSAs from EPA #5 in this context and do the following: | |
| Is able to manage a significant part of the ED (~12+ beds) or the entire ED, employing task switching in an efficient and timely manner | PC8.L4 |
| Is aware of overall changes in and flow of the ED (i.e. long triage wait times, other areas of the ED getting overwhelmed, etc.) | PC8.L4,5 |
| Has situational awareness to notice and investigate changes in their environment (i.e. hears patients fighting in the hallway or a loud crash – goes to see what is going on) | PC8.L4 |
| Is able to tailor aspects of patient workflow to the needs of the entire department, such as expediting workups when appropriate in cases of high ED volumes and long triage wait times | PC8.L5 |
| Is able to adjust individual patient management and resource utilization based on overall departmental flow when appropriate (i.e. will have ED provider spend 1 hour doing a complex facial lac when triage is empty and the ED is slow, but with 30 people in triage, consults plastics to do the facial lac) | PC8.L4 |
| Contributes to overall ED flow by reallocating resources as necessary (i.e. moves a lower acuity patient out of a monitored bed or to the hall for discharge to make room for a higher acuity patient arriving by ambulance) | SBP2.L4a |
| Is able to prioritize testing on multiple patients (i.e. who goes to CT scan first) in high volume situations or with multiple patients of high acuity | PC8.L5, SBP2.L3b,4a |
| Is able to develop or utilize solutions to bottlenecks in flow or during high volume situations | PC8.L4 |
| Is able to expedite simple patients to maximize flow of the ED | PC8.L4, SBP2.L4a |
| Effectively communicates with charge nurse and hospital administrators to optimize flow of the ED when busy | PC8.L4, ICS2.L4a/c |
| Recognizes when other providers are reaching their capacity, and intervenes to balance the workload when appropriate (if applicable), accounts for the team in decision making | ICS2.L4a |
| Is able to teach medical students and residents while managing multiple patients | PC8.L4 |
| Provides pre-hospital medical direction | ICS2.L4c |
| Effectively communicates with out of hospital personnel such as EMS / fire / police when a disaster situation arises to understand potential incoming patient burden | PC8.L4, ICS2.L4c |
| In surge situations, is able to quickly get a handle on all new patients arriving via combination of own evaluation and task delegation to other providers | PC8.L4,5 |
| When pre-notified about surge situations or mass casualty incidents, develops an appropriate plan on how to deal with and triage large numbers of patients, and activates additional resources as needed | PC8.L4,5, SBP2.L2a,3b,4c |

At which stage of training are trainees expected to achieve Level 4 entrustment: end of PGY3 year

1. **Title: Transition care to other healthcare providers.**

Specifications & Limitations: This activity includes communicating clearly, concisely and respectfully to admitting teams at transitions of care and oncoming ED providers. Using a commonly accepted structure for handoffs, and balancing being concise yet comprehensive is also included. This does not include hospital to rehabilitation or nursing home facility transfers of care.

Example Observable Practice Activities (OPAs): NA

Most Relevant Domains of Competence, including Milestone Subcompentencies that map to this EPA:

- ICS 2
- SBP1
- PC7

| **Required KSA & behaviors. To fulfill this EPA, the Trainee must:** | **Milestone Mapping** |
| --- | --- |
| **ED to inpatient handoffs** |  |
| Resident accurately and concisely articulates the reasons for admission early in the discussion | ICS2.L2,3a/c, SBP1, L3b |
| For complicated patients, resident presents in a concise manner, discussing the most urgent or significant problems first, yet ensures all important information is communicated | ICS2.L1,2,3a,3b,3c |
| Resident communicates in a collegial and respectful manner | ICS2.L1,3a,3c |
| Advocates for patients and effectively negotiates for admission or inpatient level of care when necessary | ICS2.L4b |
| Clearly states ongoing concerns about the patient, parts of workup that are not yet completed, or differential diagnoses that are still being considered but not fully investigated | PC7.L4a, ICS2.L2,3a,3b,3c, SBP1.L4c |
| Appropriately applies a standardized "transitions of care" template or methodology (if exists in institution), OR presents in an organized logical fashion | ICS2.L1,2,3a,3b,3c, SBP1.L3b |
| **ED to ED provider sign-out** |  |
| Manages transitions in care to oncoming ED providers, in a fashion that emphasizes important aspects while providing all essential information in a concise and accurate fashion | ICS2.L1,2,3b |
| During ED-ED signout, presents working dx, plan of care, unresulted tests, anticipated disposition, and IF-THEN scenarios for incomplete testing, or does this according to accepted institutional hand-off guidelines / protocol (if available) | ICS2.L1,2,3b, SBP1, L3b |

At which stage of training are trainees expected to achieve Level 4 entrustment: End of PGY2 year

1. **Title: Manage interactions with consultants**

Specifications and Limitations: This activity includes communicating with consultants respectfully, clearly and concisely, ensuring that the reason for consult is clear, the requisite information is provided and shared expectations regarding the content, process and timeline of the consult is agreed upon. Consultants must be involved at the appropriate time in the patient’s care and the resident must be able to advocate for patients when dealing with difficult consultants. The resident is also able to analyze and incorporate recommendations from the consultant to ensure the patient is receiving optimal care.

Example Observable Practice Activities (OPAs): NA

Most Relevant Domains of Competence, including Milestone Subcompentencies that map to this EPA: ICS2, SBP1/2, PROF2

| **Required KSA & behaviors. To fulfill this EPA, the Trainee must:** | **Milestone Mapping** |
| --- | --- |
| Resident accurately and concisely articulates the reasons for consultation early in the discussion | ICS2.L2,3a,3c, SBP1, L3b |
| For complicated patients, resident presents in a concise manner, discussing the most urgent or significant problems first, yet ensures all important information is communicated | ICS2.L1,2,3a,3b,3c |
| Resident communicates in a collegial and respectful manner | ICS2.L1,3a,3c |
| Avoids prematurely / inappropriately calling consults and has necessary consultant specific information ready (i.e. visual acuity before calling ophthalmology) | ICS2.L1,2,3a, SBP2.L3b |
| Recognizes when assistance with patient care is needed and involves consultants as appropriate | SBP2.L3c, PROF2.L3a, SBP1.L3c |
| Advocates for patients and effectively negotiates for consult, as well as setting expectations for consulting team | ICS2.L4b |
| Is proactive about ascertaining timeline from consulting team and obtaining recommendations in a timely fashion (when possible) | ICS2.L3a |
| Analyzes and appropriately integrates information and recommendations from consultant into optimal plan of care for patient | SBP1.L4c, SBP2.L4c |
| Uses flexible communication strategies to deal with difficult consultants | ICS2.L4b, SBP1.L4c |

At which stage of training are trainees expected to achieve Level 4 entrustment: End of PGY2 year

1. **Title: Manage complex and difficult situations**

Specifications & Limitations: This activity includes identifying, analyzing and managing difficult situations such as conflict, ethical dilemmas, and medical error disclosure including appropriately communicating with the involved parties.

Example Observable Practice Activities (OPAs) include the following types of situations:

- Ethical dilemmas
  - A conflicting advanced directive with current stated family wishes
  - A child of a Jehovah witness in hemorrhagic shock with parents refusing transfusion
  - Placing a child on a health and welfare hold due to concerns for abuse
  - Managing an impaired colleague
- Conflict
  - A high-risk patient preferring an alternate course of care than recommended such as wanting to leave AMA
  - A demanding or difficult patient (such as demanding opiates for chronic pain)
  - Disagreements between healthcare providers or team members

Most Relevant Domains of Competence, including Milestone Subcompentencies that map to this EPA:

- ICS1,2
- PROF1,2
- SBP1,2

| **Required KSA & behaviors. To fulfill this EPA, the Trainee must:** | **Milestone Mapping** |
| --- | --- |
| Ascertains all necessary information including viewpoints and concerns of patient and/or family in ethically challenging situations | ICS1.L1a/b,3a,4 |
| Involves consultants such as chaplain, social work, legal, ethics consult or other appropriate persons in difficult ethical situations | SBP1.L3c, SBP2.L2a,3b,4c, ICS2.L4c |
| Develops a well thought out and defensible plan for ethical dilemmas in complicated and challenging clinical situations | PROF1.L4b |
| “Recognizes how own personal beliefs and values impact medical care, and manages these appropriately” | PROF1.L3a |
| “Develops acceptable alternate care plans when patients’ personal decisions/beliefs preclude the use of commonly accepted practices, including high risk AMA patients” | PROF1.L3b |
| “Prioritizes the patient’s best interests” in all situations, even in challenging situations | PROF1.L4a |
| In situations where the patient is unable to speak for themselves, ascertains what the family thinks the patient would want and why | ICS1.L4 |
| “Manages medical errors according to principles of responsibility and accountability in accordance with institutional policy” | PROF2.L4b, ICS1.L4 |
| Manages conflict with patients with clear and effective communication, such as those demanding opiates, stating other seemingly unreasonable requests, or are “difficult” for other reasons | ICS1.L4  ICS1.L2a,2b,3a,4, PROF1.L1,2,3a,3b |
| Educate and counsel patient appropriately in the instance of leaving AMA, completes necessary paperwork / documentation and assess patient’s capacity to refuse recommended medical care | ICS1.L2a,2b,3a,4, PROF1.L1,2,3a,3b,4a, PC7.L3a |
| In AMA patients, understand underlying reason about why patient wants to leave and attempts to find a collaborative mutually acceptable solution with patient | PROF1.L1,2,3a,3b,4a ICS1.L1a,1b,2a,3a,4a |
| Manages conflict between healthcare personnel or team members, such as nurse-physician conflict | ICS2.L4b |
| “Can form a plan to address impairment in one’s self or a colleague, in a professional and confidential manner“ | PROF2.L4a |

At which stage of training are trainees expected to achieve Level 4 entrustment: end of PGY3 year

1. **Utilize recommended patient safety and quality improvement processes**

Specifications & Limitations: This activity includes utilizing existing hospital processes and technologies to maximize current and future patient safety and quality. This does not include developing and implementing one’s own quality improvement project.

Example Observable Practice Activities (OPAs): NA

Most Relevant Domains of Competence, including Milestone Subcompentencies that map to this EPA: SBP1,3, PBLI

| **Required KSA & behaviors. To fulfill this EPA, the Trainee must:** | **Milestone Mapping** |
| --- | --- |
| “Employs processes (i.e. checklists, order sets) that optimize patient safety” and quality | SBP1.L3b, PBLI.L4a |
| “Employs personnel that optimize patient safety and quality (i.e. ED pharmacists) | SBP1.L3b |
| Utilizes technology such as order sets and “decision support systems within the EHR” (i.e. allergy alerts for medication ordering, embedded CDRs) to optimize patient safety and quality | SBP1.L3b, SBP3.L4, PBLI.L4a/c |
| *Utilizes hospital event reporting system and other related processes / technologies in order to optimize future patient safety and quality* | SBP1.L3b, PBLI.L4a |
| “Participates in institutional process improvement plans to optimize ED practice and patient safety” | SBP1.L4a, PBLI.L4a/c |
| *Identifies and communicates elements of patient care processes in which changes could result in improved patient quality or safety, such as latent system errors* | SBP1.L4c |
| Analyzes their own cases to improve their own and others performance | SBP1.L4b |

At which stage of training are trainees expected to achieve Level 4 entrustment: end of PGY2 year

Legend: Linked milestones are indicated using the following format – Subcompetency (PC1).Proficiency Level (Lx).a/b/c/d. The lowercase letters represent the individual milestones listed within each proficiency level column on the ACGME/ABEM Milestones document, with the first milestone listed in each column being denoted as “a”, the second one as “b”, etc. For example, the 3^rd^ milestone under Patient Care 1, Proficiency level 3 would be PC1.L3c. Any italicized KSABs are less common occurrences, which individual programs one might elect to assess outside of this EPA framework. The KSABs in quotes are taken directly from the Emergency Medicine milestones.^2^ NA = Not applicable, this is entered when an item does not link directly to a milestone item. Core competency / subcompetency abbreviations are as follows: PC = patient care, MK = medical knowledge, ICS = interpersonal communication skills, PROF = professionalism, PBLI = problem based learning and improvement, SBP = system based practice.
