# Supplementary material for: Integration of Entrustable Professional Activities with the Milestones for Emergency Medicine Residents
Source: West J Emerg Med. 2018 Nov 30;20(1):35–42. doi: 10.5811/westjem.2018.11.38912 (PMC6324698; doi:10.5811/westjem.2018.11.38912)
Supplement: Supplementary file 2 [file wjem-20-35-s002.docx]

**Appendix 2.** Requisite characteristics & competencies to all entrustable professional activities.

| Ten Cate’s general conditions for trust | |
| --- | --- |
| Integrity | “Benevolence: having favorable intentions, honesty, and truthfulness “   - Acts responsibly, is truthful and accountable for actions and words |
| Reliability | “Working conscientiously and showing predictable behavior “   - - Trainee is thorough and accurate in data gathering   - Trainee is dependable in following through with assigned or necessary tasks |
| Humility | “Discernment of own limitations and willingness to ask for help when needed”   - - Recognizes limits of their own knowledge and skill in all situations, and asks for help when needed   - Uses just-in-time resources to access medical information when additional knowledge is required |
| Additional characteristics and competencies required for trust | |
| Respectfulness | - - Trainee is respectful of patients, including those from diverse backgrounds   - Trainee is respectful of all members of the healthcare team, including paramedical staff   - Values and receives questions / input / suggestions from all members of the healthcare team (specifically nursing) in a collaborative manner |
| Self-monitoring and resilience | - - Trainee demonstrates readiness for work; recognizes when outside occurrences are affecting their attitude or performance, and minimizes this influence   - Identifies when elements of burnout or work-related stress (compassion fatigue, depersonalization, emotional exhaustion, cynicism) is affecting their attitude or performance, and minimizes this influence |
| Self-assessment and self-improvement | - - Trainee recognizes, reflects upon and elucidates (via feedback from others) own strengths and weaknesses and makes improvements in performance accordingly   - Demonstrates accurate self-reflection skills |
